# Education in the Anthropocene: assessing planetary health science standards in the USA

**DOI:** 10.1098/rspb.2023.0975

**Published:** 2023-09-27

**Authors:** Samantha L. R. Capel, Brian F. Allan, Alonso Favela, C. Scott Clem, Sean Khan Ooi, Stephany Virrueta Herrera, Loralee J. Wilson, Lynette R. Strickland

**Affiliations:** ^1^ Wildlife Health Laboratory, California Department of Fish and Wildlife, Wildlife Genetics Research Unit, Sacramento, CA 95834, USA; ^2^ Department of Evolution, Ecology, and Behavior, University of Illinois Urbana-Champaign, Urbana, IL 61801, USA; ^3^ Department of Entomology, University of Illinois Urbana-Champaign, Urbana, IL 61801, USA; ^4^ Program in Ecology, Evolution, and Conservation Biology, University of Illinois Urbana-Champaign, Urbana, IL 61801, USA; ^5^ School of Plant Sciences, University of Arizona, Tucson, AZ 85721, USA; ^6^ Department of Entomology, University of Georgia, Athens, GA 30602, USA; ^7^ Department of Biology, Boston University, Boston, MA 02215, USA

**Keywords:** USA Science Education, planetary health, K-12 science standards, global change

## Abstract

The environmental crises defining the Anthropocene demand ubiquitous mitigation efforts, met with collective support. Yet, disengagement and disbelief surrounding planetary health threats are pervasive, especially in the USA. This scepticism may be influenced by inadequate education addressing the scope and urgency of the planetary health crisis. We analysed current K-12 science standards related to planetary health throughout the USA, assessing their quality and potential predictors of variation. While planetary health education varies widely across the USA with respect to the presence and depth of terms, most science standards neglected to convey these concepts with a sense of urgency. Furthermore, state/territory dominant political party and primary gross domestic product (GDP) contributor were each predictive of the quality of planetary health education. We propose that a nation-wide science standard could fully address the urgency of the planetary health crisis and prevent political bias from influencing the breadth and depth of concepts covered.

## 1. Introduction

Global environmental change due to overpopulation, overexploitation of resources, climate change, biodiversity loss and interrelated factors endanger the future of humanity [1,2]. Their effects include food and water crises, health declines and increasing rates of natural disasters [3–5]. Moreover, these effects are disproportionately pronounced for impoverished and underprivileged communities [6–8] and will continue to intensify in magnitude and inequity without rapid, ubiquitous intervention [9]. Mitigating the catastrophic impacts of anthropogenic planetary change will require informed, urgent and collective action. Hence, comprehensive education of concepts surrounding human influences on the biosphere is crucial, especially in countries which have the largest influence on the biosphere, such as the USA [10]. Without proper understanding of global health crises, disengagement and disbelief surrounding planetary health threats ensue, posing serious barriers to solutions as these represent key predictors of individual and collective action [11]. Adequate public school education on planetary health topics offers a critical tool to promote widespread support for mitigative action by informing citizens [12], fostering climate change concerns among parents [13] and ultimately stimulating collective sustainable behaviours [14,15].

The nascent field of planetary health seeks to address the consequences of anthropogenic environmental change for ‘the health of human civilization and the state of the natural systems on which it depends' [16]. While many universities in the USA offer curricula on planetary health and related subjects, only a minority of USA citizens (34.6%) attain a bachelor's degree, whereas most (89.7%) successfully complete secondary education [17]. Though education on planetary health topics like climate change has mixed results among adults [18], education fosters concern and mitigation behaviours in adolescents [19], which can positively influence parental beliefs and actions [13]. Moreover, universally accessible education will empower and reduce inequities for communities most affected by planetary health threats [6,8] and achieve solidarity from those who are less impacted [20].

It is essential that every student receives an unbiased education on planetary changes that characterize the Anthropocene. Therefore, teaching a comprehensive and frequently renewed understanding of these subjects in public schools should be an objective of the USA education system. Several studies have sought to assess if and to what degree subjects related to planetary health are being taught in USA public schools. Research evaluating how topics such as evolution [21], sustainability [22], ecology [23] and global climate change [24] are portrayed in state standards and curricula identify high variation among states in how thoroughly concepts are portrayed and discussed. In one study of high school textbooks, the language used to describe research on climate change was found to be vague and often contained no explicit cause-effect language that connected human activities with climate change [25]. In many cases, scientists' views on human-induced climate change are framed as controversial or portrayed with uncertainty and doubt. A set of white papers recently produced by the National Center for Science Education reviewed how climate change is addressed in all 50 state science standards, and found that some standards even ask students to ‘debate the issue', serving as a means to bring non-evidence-based perspectives into science classrooms [26]. Similarly, work assessing how specific topics such as sustainability and the environment are portrayed in the Next Generation Science Standards (NGSS), a set of national standards adopted by approximately one-third of all states and territories, identified abstract language that portrayed the environment as a loosely defined entity rather than an interconnected set of complex biological systems which include humans [27,28]. Together, these studies call into question the quality and consistency of sustainability-focused science education in the USA and highlight the need for further investigation.

Here, we evaluate the capacity of the USA K-12 public education system to prepare students to understand, cope with, and help mitigate the current trajectory of planetary health. Using a comprehensive list of major concepts and issues, we gauged the scope of planetary health education in USA science standards. We measured planetary health education quality using key terms indicative of planetary health concepts for all USA state/territory science standards based on: (i) the depth of term presence, (ii) the degree to which terms are described as having anthropogenic causes and/or effects (i.e. human interactions), and (iii) the level of urgency presented with relevant terms. While variation in language use surrounding planetary health concepts in education standards have previously been assessed on a nation-wide scale [24,29], little attention has been paid to attempt to identify potential state-level political and economic drivers of this variation. Here, we used measures of state dominant political party and economic factors to identify state characteristics predictive of planetary health education quality across the USA. We also evaluated how the NGSS performed in comparison to those developed by individual states.

## 2. Methods

### (a) State science standards and metadata

We evaluated state science standards for USA public education as of July 2020 for the presence and framing of topics related to planetary health. Standards provide a crucial backbone for which teachers, textbook publishers, standardized test makers and others use to establish education goals [30]. While most states and territories adhere to their own standards, 17 states, 1 USA territory and Washington D.C. have fully adopted the NGSS; a set of national standards meant to improve and unify USA science education developed by Achieve, a nonprofit education organization, in collaboration with the National Research Council (NRC) and other partners [31]. The current science standards for all states and territories for which standards could be located (i.e. American Samoa, Guam and Puerto Rico), as well as Washington D.C., were compiled and categorized into those that use NGSS and those that follow their own standards. States were also characterized by the dominant political party of their state legislature the year of science standard adoption [32]. Further, we recorded the geographical region [33], major economic industry as determined by the primary GDP contributor [34], level of climate change preparedness [35] and average household income [36] for each state and territory. All science standards, raw data and code are available via Dryad (doi:10.5061/dryad.rn8pk0phr).

### (b) Assessment terms and dimensions

All state-level standards, including NGSS, were evaluated for five fundamental concepts and 10 major issues chosen based on terms we found to be critical for a comprehensive understanding of planetary health and the current trajectory of the global climate crisis following a review of recent literature published on sustainability [37], climate change impacts [3] and biodiversity loss [38–40] (electronic supplementary material, table S1). Terms were searched throughout the standard to identify the most descriptive text segments (sentences and paragraphs) associated with the topic and were ranked based on three separate categories: (i) Term Presence, (ii) Human Interaction and (iii) the Level of Urgency conveyed within standards (electronic supplementary material, table S2), hereafter referred to as ‘dimensions'. We used ‘Term Presence' to assess the level to which each search term or phrase was presented within science standards. This dimension was scored from 0 to 3, representing ‘absent' (0), ‘indirectly mentioned' (1), ‘briefly mentioned’ (2) and ‘described in-depth' (3). We assessed ‘Human Interactions' which measured the degree to which each term was conveyed as being affected by humans and/or affecting humans. This dimension was also scored from 0 to 3 based on the level of connectedness; ranging from: ‘absent' (0), ‘indirect' (1), indicating an indirect or implied connection to humans, ‘unidirectional' (2), denoting that the standard explicitly tied the term or phrase to affecting humans or being affected by humans (but not both), and ‘bidirectional' (3), indicating the term or phrase was both affected by and affecting humans. Lastly, we evaluated ‘Level of Urgency' which quantified how pressing or critical the term was conveyed based on language that indicated urgency or threat to human well-being. This dimension ranged from 0 to 2, with scores representing ‘absent' (0), ‘moderate or implied' (1), and ‘high' (2). Examples of language indicative of each rank can be found in electronic supplementary material, §1. The five foundational concepts (ecology, evolution, biodiversity, ecosystem and ecosystem services) were not assessed for Level of Urgency, as this dimension was not relevant for these terms. For clarification on the methodological nomenclature used, refer to electronic supplementary material, §2.

### (c) Reviewer assessment and statistical analyses

The education standards for each state were assessed by three separate, randomly assigned reviewers. Randomized assignment of state education standards was performed using the RANDARRAY function in Excel [41]. Reviewers assessed each state education standard in its entirety as it related to biology, including general biology, Earth and planetary sciences and environmental sciences from each state. For NGSS, both the published standards as well as the NRC's K-12 Framework for Science Education from which they were developed [42] were assessed. Final ranks for each term within each standard were calculated as the average rank of the three reviewers. To account for differences between reviewers for Term Presence, the variance was calculated and any ranks with a variance of 0.667 or higher were re-evaluated by the reviewers. Mean dimension scores (i.e. Mean Term Presence Score, Mean Human Interactions Score and Mean Level of Urgency Score) were calculated for each standard as the per cent of the total possible sum of all ranks within each dimension across all terms. Composite scores were also calculated for each standard as the average of all mean dimension scores (see electronic supplementary material, table S3 and §3 for more details). Mean dimension scores as well as composite scores were also calculated for each term across all unique standards (i.e. all NGSS states considered as an individual standard; electronic supplementary material, table S4).

We performed restricted maximum-likelihood linear mixed models, using the statistical package *asreml-r* in r version 4.0 [43], to determine which state characteristics were predictive of variation in mean dimension scores among individual state and territory standards. Modelling was done iteratively, first with all hypothesized predictive factors run individually as a fixed factor, while all other factors were treated as random factors. Wald's significance tests were performed on individual models to identify factors that significantly predicted mean dimension scores (see electronic supplementary material, table S5 for model details).

## 3. Results

### (a) Planetary health education among states and territories

**Figure 1.**
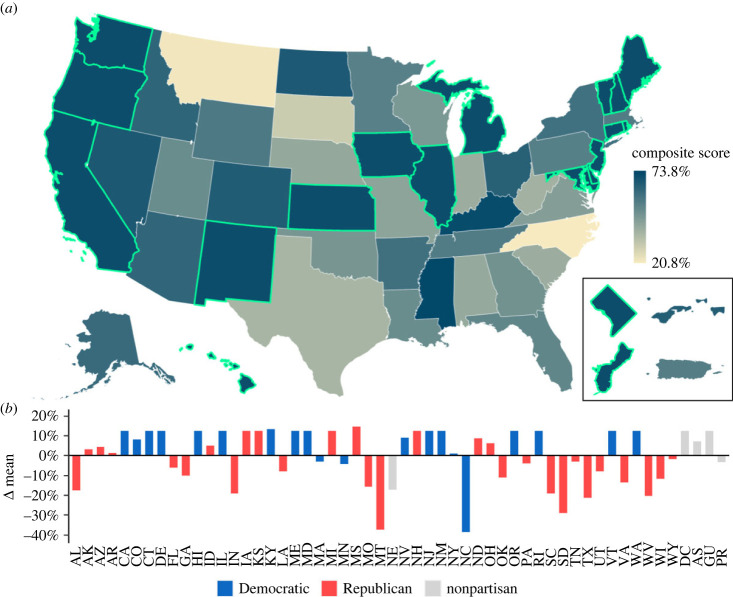
Planetary health education quality across individual state/territory standards. Educational science standard quality across the United States (*a*) is represented by each state/territory composite score. The inset box shows composite scores for Washington, D.C. (top left; DC), American Samoa (top right; AS), Guam (bottom left; GU) and Puerto Rico (bottom right; PR). States and territories that have fully adopted NGSS are outlined in bright green. The deviation from the mean for each state/territory science standard composite score (*b*) is shown along with state dominant political party.

**Figure 2.**
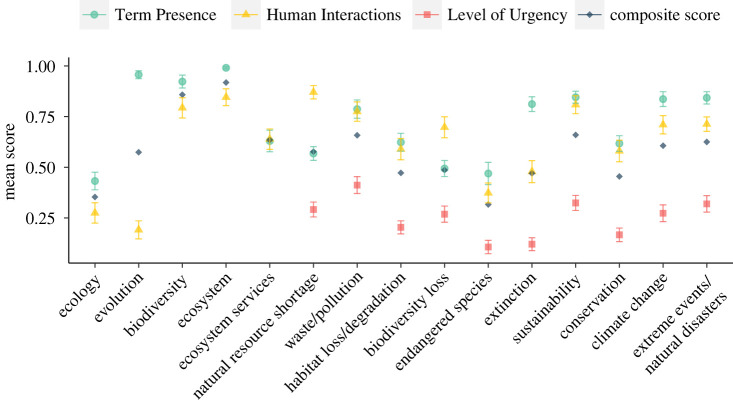
The distribution of planetary health education quality across all terms and dimensions. Performance of individual terms for Term Presence (teal circles), Human Interactions (yellow triangles) and Level of Urgency (red squares) is represented by the mean dimension score for each term across all standards and the associated standard error. Also shown is the composite score for each term (dark blue diamonds). All states and territories that adopted NGSS in full were considered as a single standard.

Composite scores for state/territory science standards varied widely (figure 1*a*; electronic supplementary material, table S3), ranging from 20.8% (North Carolina) to 73.8% (Mississippi). Composite scores for terms (figure 2; electronic supplementary material, table S4) also exhibited high variance, ranging from 31.6% (endangered species) to 91.8% (ecosystem). Mean dimension scores for state standards showed strong positive correlations (electronic supplementary material, figure S1), with Term Presence and Human Interactions showing the strongest correlation (adjusted *R*^2^ = 0.671), followed by Human Interactions and Level of Urgency (adjusted *R*^2^ = 0.625), and finally Term Presence and Level of Urgency (adjusted *R*^2^ = 0.480). These correlations between the three dimensions suggest that the inclusion of a term within a science standard also indicates that the term is more likely to be presented with some degree of human interconnection and urgency. While any given individual dimension is thus informative, considering all three dimensions together provides a measure of the overall quality of planetary health education. Tests for significant interactions between mean scores and standard lengths indicated no significant relationships (electronic supplementary material, figure S2).

### (b) Interrater reliability

We assessed interrater reliability using Krippendorff's *α* with data defined as ‘ordinal'. Bootstrapped values were calculated using 20 000 replicates along with 95% confidence intervals using the icr r package [44]. Interrater reliability was high, with a 95% confidence interval between 0.87 and 0.89 (electronic supplementary material, figure S3).

### (c) Trends across states and territories

Across all terms and individual state/territory standards, Term Presence was moderately high (figure 2; electronic supplementary material, figure S4), with approximately half of the terms being at minimum briefly mentioned (8 out of 15 scored ≥ 66.7%; mean = 72.2%). However, most standards did not describe terms in-depth, and only six had a mean Term Presence score above 83.3% (i.e. on average, more than half of the terms were described in-depth). Human Interactions scores were generally lower (mean = 62.3%) than for Term Presence, still about half of the terms were described as having either direct anthropogenic causes or effects (8 out of 15 scored ≥ 67%). The five foundational concepts had higher Term Presence than the 10 major issues (mean = 78.7% versus 69.0%) while the reverse was true for Human Interactions (mean = 54.9% versus 66.0%).

From our analyses, it is apparent that most concepts required for understanding planetary health challenges lacked language that conveyed urgency. Across all standards and terms, the average Level of Urgency score was 24.9%, the lowest among dimension mean scores (electronic supplementary material, table S4 and figure S4). The terms conservation, extinction and endangered species were associated with the lowest Levels of Urgency (respective mean; 16.7%, 12.0% and 10.6%) while waste/pollution received the highest mean score (41.2%). Many standards associated no urgency with major issues, with 123 of the total 360 terms across all standards receiving a Level of Urgency rank of 0 (electronic supplementary material, figure S4).

### (d) Determinants of science standard scores

As major economic industry and dominant political party strongly influence individuals' views on planetary health [45], another goal of our study was to assess whether these factors play a role in driving differences in planetary health education among USA states and territories. Using restricted maximum-likelihood linear mixed models, we examined whether state dominant political party, economic industry, climate change preparedness, average household income or geographical location were significant predictors of mean dimension scores.

**Figure 3.**
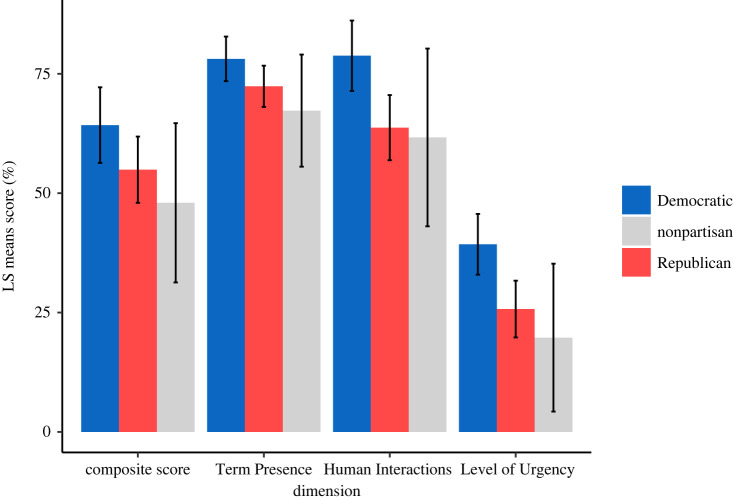
Political influence on state/territory science standards for planetary health education. Represented by least-squares (LS) means and the 95% CI of dimension scores across state/territory dominant political party the year state standards were adopted. LS-means and significance were calculated using a linear mixed effects model. Term Presence was not significantly correlated with the dominant political party of the state at time of implementation (Wald's test*: p* = 0.09), whereas Human Interactions and Level of Urgency were significantly correlated with dominant political party at the time of state standard implementation (Wald's test*: p* < 0.005). Nonpartisan states show the greatest degree of standard error of prediction (statistical model and results presented in electronic supplementary material, table S5).

Dominant political party was the strongest predictor of the quality of planetary health education across most ranking metrics (figure 3). While it did not predict Term Presence (*p* = 0.09), it did predict differences in Human Interactions and Level of Urgency (*p* < 0.005; electronic supplementary material, table S5). We found that Democrat-led states received predicted mean scores 18% higher for Human Interactions and 33% higher for Level of Urgency than Republican-led states. Nonpartisan states (*n* = 5) exhibited the largest degree of variation in prediction and typically had the lowest predicted mean compared with Republican- and Democrat-led states. Of the states which scored below the mean composite score (i.e. 59.3%), 86% were Republican-led states while 14% were Democrat-led (figure 1*b*). This trend is partially explained by the proportion of Democrat-led states that have adopted NGSS (65%) versus Republican-led states (14%), given that NGSS received the third highest composite score.

**Figure 4.**
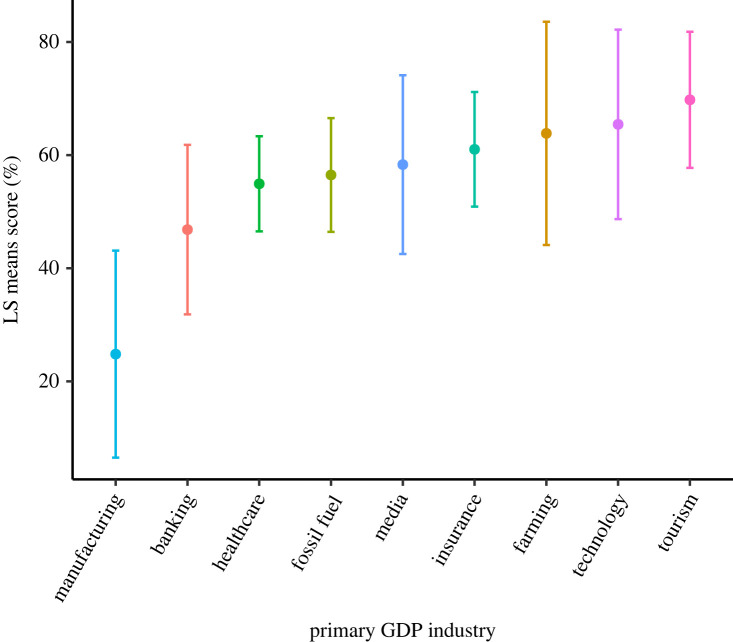
Dominant state industry influence on state/territory science standards for planetary health education. Least-squares means, and the 95% CI calculated from the ASREML-r model presented in electronic supplementary material, table S5. Figure highlights how state/territory science standard composite scores are predicted by largest contributor to GDP. Largest GDP industry of state was determined from data collected by the Bureau of Economic Analysis.

Aside from dominant political party, state/territory major economic industry was the only other significant predictor of the quality of planetary health education (figure 4; electronic supplementary material, table S5). States and territories with economies dominated by agricultural industries had the highest predicted composite score (69.8%), while states with major manufacturing industries had the lowest predicted composite score (25.9%). Interestingly, it appears that states with industries that are dependent on environmental conditions (e.g. agriculture and tourism) have higher predicted composite scores (68.0%) compared with those with industries that are not dependent on environmental conditions (42.9%) (e.g. manufacturing and fossil fuels).

## 4. Discussion

We investigated whether topics essential for developing a robust understanding of the challenges of the Anthropocene are present in K-12 science standards across USA states and territories. We found that many topics are present in science standards and conveyed with conceptual depth. However, we found that nearly all major issues of planetary health assessed here are severely lacking any sense of urgency within science standards. Moreover, there was significant variation in the quality of planetary health education across USA states and territories, with dominant political party (i.e. Republican- versus Democrat-led) being the strongest predictor of science standards depth and breadth. While decades of research and discussion have implicated schools as a form of social control and education as inherently political [29], to our knowledge, we are the first study to statistically quantify and connect state dominant political party with variation in the depth and breadth of planetary health education in USA public schools and identify additional state characteristics (i.e. dominant economic industry) associated with planetary health education.

The lack of urgency surrounding aspects of planetary health education across the USA has been noted previously, particularly in reference to climate change [25]. Vague language and a lack of concrete discussion on the implications of global change leave students ill-equipped to cope with and mitigate threats to their health and livelihood. At present, a large proportion of the USA population, including both children and adults, fail to recognize the inextricable connections between sustainable ecosystems, human health and ultimately societal stability [46]. Neglecting to explicitly define the anthropogenic connections of planetary health and the urgency of its current prognosis does little to convey the deep significance human actions play in the sustainability of our own livelihoods and those of future generations. Our emphasis on urgency seeks to foster optimistic mitigatory and adaptive action within the USA.

Perhaps one explanation for the variation in how major planetary health issues in USA science standards are conveyed is political influence on standard development and/or implementation. Our findings suggest that partisanship of state and territory legislatures influence the quality of planetary health education in public school science standards. This relationship is likely a direct result of the heavy politicization of issues like climate change influencing state officials involved in the adoption of state-specific legislation, including public school education standards [47–49]. In addition to trends in planetary health education between Democrat- and Republican-led states, we observed possible indications of political influence in the process of education standard adoption in the language of several standards. For example, the South Dakota science standards stated that ‘not all viewpoints can be covered in the science classroom' when referencing climate change and evolution and requested that ‘parents engage their children in discussions' in order to allow students to ‘draw their own conclusions'. The current process of standard adoption can result in politically biased standards influenced by non-experts, as has been seen in other science topics such as evolution [30]. Non-science-based political views extending into the classroom and thus shaping the beliefs for millions of Americans is a failure of the public education system. Politicization of scientific topics can drive science scepticism and in turn directly impact individual behaviour, often to the detriment of public health, a phenomenon which has contributed to the severity of the COVID-19 health crisis [50]. Removing measurable political bias from state science education standards is essential for addressing and surviving the challenges posed by the Anthropocene.

Economic industries unexpectedly appear to influence K-12 planetary health education standards. Our findings indicate that the major economic industry of the state (as characterized by GDP) was significantly associated with planetary health education quality (figure 4). Interestingly, there appears to be little research assessing the influence of major economic industry on state-level climate change education. However, studies have explored how industries' internal policies respond to the threat of climate change [51] namely, finding that most organizations select the path that is most like the *status quo* or ‘business as usual', avoiding incorporation of more sustainable practices. Perhaps this trend explains the correlation between industry and education standards we observed, with states that benefit economically from maintaining *status quo* resisting higher-quality standards. Further, it is well known that industries differ in their vulnerability to climate change [52–55] and that specific occupations can shift ecopsychological views to be more concerned about climate change [56–59]. Hence, policymakers in states with environmentally dependent industries (e.g. farming and tourism) may be more aware of the environmental degradation caused by these systems, which may in turn foster support for more comprehensive planetary health education. Alternatively, the negative environmental impacts of industries such as farming and technology may simply be more apparent to local educators, motivating them to incorporate more extensive planetary health standards. This apparent association between dominant economic industry and planetary health education warrants further investigation to determine the causal nature of the relationship.

As many Americans do not receive institutional education beyond high school [17], we cannot rely on universities to disseminate the cutting-edge knowledge of the impacts of anthropogenic environmental change. In order to better serve all students, we suggest enacting a unified science education standard across the USA which not only encompasses topics necessary for a comprehensive understanding of planetary health, but also presents them with the appropriate level of urgency while negating partisan influence of politicized issues. We maintain that ubiquitous adoption of the NGSS would be the best first step towards achieving this goal. In this study, NGSS had the third highest composite score among individual standards, ranking first in Human Interactions, fourth in Level of Urgency and sixth in Term Presence, and is therefore one of the highest performing standards in this analysis. NGSS was developed by a team of administrators, educators and researchers from 26 states evenly representing both major political parties (14 Democrat-led, 12 Republican-led) [22]. We found that topics that have been heavily politicized (e.g. evolution, climate change and sustainability) were presented in-depth and regularly shown to have human interconnections. Currently, 19 states and territories have adopted NGSS in full and another 13 directly reference them in their standards. Thus, full adoption of NGSS by the remaining 35 states and territories could curtail politicization of science concepts in addition to increasing planetary health education consistency and quality across the nation. We acknowledge that every state faces unique environmental challenges that warrant attention in science standards and that universal adoption of NGSS may seem unappealing to those states that have developed learning objectives dedicated to local issues (e.g. New York and Florida, among others). However, rather than hindering education on state-specific topics, we propose that adoption of NGSS would allow state-level educators to devote more resources to developing supplements dedicated to local issues.

While NGSS would serve as a suitable starting point for a universal science standard, this analysis indicates it is still wanting in setting requirements for comprehensive planetary health education. We are not alone in this finding; a report released by the National Center for Science Education and the Texas Freedom Network Education Fund in 2020 identified similar trends among climate-change-related USA science education standards [26]. Specifically, both this study and the NCSE report assigned the highest score/grade to states that have not adopted NGSS; Mississippi in this study and Wyoming, Alaska, Colorado, New York and North Dakota in the NCSE report. These findings suggest that several states have incorporated a more thorough discussion of topics related to planetary health within their standards and may serve as examples for improving NGSS. The composite score for NGSS was 71.8%, indicating a need for improvement on the topics assessed, particularly in relaying urgency (mean Level of Urgency score was 45%). Topics such as habitat loss/degradation, endangered species and extinction had the lowest scores for this dimension in both NGSS and non-NGSS standards, indicating that these topics are not being presented with the urgency necessary for the alarming rates at which they are occurring. Moreover, the framework from which current NGSS standards were developed was published in 2011, meaning information on topics that are subject to very active research, such as climate change and biodiversity loss, requires frequent updating. The pace of planetary health degradation and coinciding research calls for updated standards and a reform process that functions at a similar pace. We suggest implementation of systems to allow for efficient dissemination and incorporation of up-to-date planetary health findings into K-12 science education standards. Enacting universal science standards would facilitate this process as the task of updating standards would be centralized to a single entity rather than dispersed among legislatures, ensuring that students who are expected to prosper in the Anthropocene will be literate in the planetary health issues they will face.

As we have focused solely on state-level science standards, we acknowledge that they do not necessarily dictate classroom curricula across all districts. Some teachers may go beyond state standards regarding the topics under evaluation as a result of well-funded districts, ample professional development opportunities and/or personal values. Teachers that lack resources in their districts may not have the means to meet standards, regardless of their desire to produce well-prepared students. Educators may also face hostile local communities, causing them to avoid discussing potentially controversial topics in the classroom such as climate change, planetary health and evolution. Moreover, internal bias among teachers may influence how these topics are framed in the classroom or if they are even discussed at all [30,60]. Systemic solutions such as a mandated, universal science standard would promote equity among teachers in their ability to comprehensively educate students on planetary health topics by obligating districts to adopt curricula that meet those standards. Doing so may also help bridge the gap in planetary health education between K-12 education and post-secondary institutions and promote awareness and activism among all students regardless of their level of education.

Enacting a unified science standard across the USA will not be a simple task. There is a great deal of work that goes into the development of standards and requires collaboration between educators, policymakers and scientists [61]. Additionally, the quick and efficient implementation of updated standards would require educators to have access to professional development tools and training needed to teach novel curricula and best serve their students. While enacting this strategy may be a large undertaking, it would better equip students to deal with massive global issues which will need to be addressed within the next decade to truly affect current global trajectories [62]. Once a leader of environmental policies and initiatives, many of the first environmental movements took place in the USA in response to intense industrialization and exploitation of natural resources in the nineteenth century [63]. Today however, the USA lags behind China and India in renewable energy investment and falls far behind other developed nations including Iceland, Denmark, Norway and France in establishing and meeting climate change initiatives [64,65]. The underperformance of the USA response to climate change could very well be connected to the lack of consistent and comprehensive planetary health education across the nation. The unrelenting pace of global change has created unprecedented urgency for all corners of society to adapt, especially in the field of education [66–68]. While we cannot speak to global trends, assessing planetary health education in the world's second largest greenhouse gas emitter [69] is imperative for anticipating trends in the success of sustainability practices. We affirm that comprehensive, accessible and appropriately urgent public education on planetary health is indispensable for confronting the environmental challenges to come. While the task of updating science standards to keep pace with the current trajectory of planetary change is daunting, we hope that our findings will identify a way forward and begin conversations for decisive action.

## Data Availability

Data available from the Dryad Digital Repository: https://doi.org/10.5061/dryad.rn8pk0phr [70]. Supplementary material is available online [71].
